# HGF/c-Met Axis: The Advanced Development in Digestive System Cancer

**DOI:** 10.3389/fcell.2020.00801

**Published:** 2020-10-26

**Authors:** Zhiwei Shao, Haoqi Pan, Sheng Tu, Jingying Zhang, Sheng Yan, Anwen Shao

**Affiliations:** ^1^Department of Hepatobiliary and Pancreatic Surgery, Second Affiliated Hospital, School of Medicine, Zhejiang University, Hangzhou, China; ^2^Department of General Surgery, Sir Run Run Shaw Hospital, School of Medicine, Zhejiang University, Hangzhou, China; ^3^State Key Laboratory for Diagnosis and Treatment of Infectious Diseases, Collaborative Innovation Center for Diagnosis and Treatment of Infectious Diseases, The First Affiliated Hospital, College of Medicine, Zhejiang University, Hangzhou, China; ^4^Department of General Surgery, Second Affiliated Hospital, School of Medicine, Zhejiang University, Hangzhou, China; ^5^Department of Neurosurgery, Second Affiliated Hospital, School of Medicine, Zhejiang University, Hangzhou, China

**Keywords:** hepatocyte growth factor, c-Met, molecular targeted therapy, digestive system cancer, inhibitors

## Abstract

Numerous studies have indicated that abnormal activation of the HGF/c-Met signaling pathway can lead to cell proliferation, invasiveness, and metastasis of cancers of the digestive system. Moreover, overexpression of c-Met has been implicated in poor prognosis of patients with these forms of cancer, suggesting the possibility for HGF/c-Met axis as a potential therapeutic target. Despite the large number of clinical and preclinical trials worldwide, no significant positive success in the use of anti-HGF/c-Met treatments on cancers of the digestive system has been achieved. In this review, we summarize advanced development of clinical research on HGF/c-Met antibody and small-molecule c-Met inhibitors of cancers of the digestive system and provide a possible direction for future research.

## Introduction

Cancers of the digestive system comprise hepatocellular carcinoma (HCC), gastric, pancreatic, esophageal, and colorectal cancers (CRCs) and have been reported to result in high morbidity and mortality all over the world (Zhang H. et al., 2018). A number of intervention methods including surgical treatment, chemotherapy, radiotherapy, and targeted drug treatment have been tried, but their overall effect on managing cancers of digestive system is still unsatisfactory (Ang et al., 2016). During advanced stages of development, systemic therapies based on targeted molecular drugs and cytotoxic chemotherapy holds the most promise to successful treatment of these cancers. For instance, rat sarcoma (RAS)-wild-type CRC was successfully treated with epidermal growth factor receptor (EGFR) monoclonal antibodies while human epidermal growth factor receptor 2 (HER2) high expression gastric cancer was managed in patients using the HER2 monoclonal antibody trastuzumab (Van Schaeybroeck et al., 2011; Gomez-Martín et al., 2014). Studies have shown that the hepatocyte growth factor (HGF)/mesenchymal epithelial transition (c-Met) signaling pathway plays a key role in growth, invasiveness, metastasis, and acquired drug resistance of cancers of digestive system. Based on this evidence, inhibition of HGF/c-Met axis may be a promising and revolutionary treatment method for these conditions.

## HGF/C-Met Signaling Pathway

**FIGURE 1:**
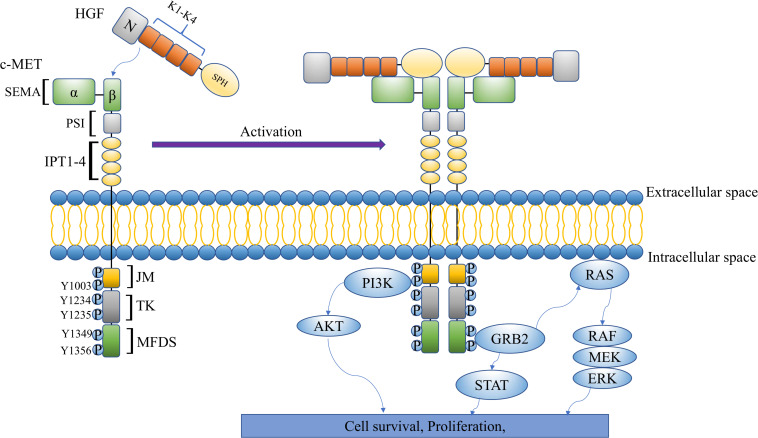
The multidomain structure and signaling pathway of HGF/c-Met.

The c-Met proto-oncogene was originally identified in human osteosarcoma cells in the 1980s. It is located on chromosome 7q21-q31, encoding receptor tyrosine kinase c-Met (Cooper et al., 1984). Various differences between c-Met and other receptor tyrosine kinases can be found in the structure. Particularly, it is a heterodimer composed of a highly glycosylated 50-kDa α subunit and a transmembrane 145-kDa β subunit that are connected by a disulfide bond (Crepaldi et al., 1994). The basic structure of c-Met includes three parts: an extracellular structure, an intracellular structure, and a transmembrane domain. The extracellular part of c-Met is composed of a big N-terminal Sema, plexin-semaphorin-integrin (PSI), as well as four immunoglobulin-like IPT domains (IPT1–4) (Gherardi et al., 2006). On the other hand, the intracellular part is mainly composed of a juxtamembrane domain (JM), ser975, that plays a negative regulatory role in the signaling pathway, and a Y1003 residue that regulates degradation (Peschard et al., 2001). In addition, the Y1234 and Y1235 residues constitute the catalytic domain of c-Met where they positively regulate the pathway (Bradley et al., 2018). HGF is also located on chromosome 7q21.1 and is the only known c-Met ligand with high affinity (Parikh and Ghate, 2018). Mature HGF is an α*-*β heterodimer linked by disulfide bond. The α-chain (69 kDa) comprises an N-terminal (N) hairpin-loop and 4 kringle domains (K1–K4) while the β-chain (32–34 kDa) has a serine proteinase homology (SPH) domain (Holmes et al., 2007). It has been reported that autophosphorylation of Y1234 and Y1235 triggers further autophosphorylation of Y1349 and Y1356 residues in the multifunctional docking site (MFDS) (Miranda et al., 2018). The autophosphorylation of tyrosine residues can activate a multisubstrate docking site that comprises a variety of intercellular factors including Grb2-associated binding protein 1 (GAB1), Grb2-associated binding protein 1 (GAB2), phospholipase C (PLC), and sarcoma (SRC). With these, it can attract SH2 domain-containing tyrosine phosphatase 2 (SHP2), CT10 regulator of kinase-homolog-like (CRKL), and other docking molecules to activate Ras/mitogen-activated protein kinase (MAPK), PI3K/anaplastic lymphoma kinase (ALK), signal transducer and activator of transcription (STAT), and other signaling pathways (Eder et al., 2009; Gherardi et al., 2012). Activation of downstream signaling leads to regulation of cell proliferation, invasiveness, metastasis, and angiogenesis (Rong et al., 1994; Bladt et al., 1995; Michalopoulos and DeFrances, 1997; Xu et al., 2018; Figure 1).

## Deregulation of HGF/C-Met Mediates Cancer

The strict regulation of HGF/c-Met signal transduction, observed in growth and regeneration, results in different degrees of maladjustment in various cancers, especially in the case of drug resistance or metastasis (Bradley et al., 2018). Failure to regulate the pathway leads to protein overexpression or, amplification, as well as gene mutations. In addition, a crosstalk between HGF/c-Met and other signaling pathways and increased paracrine and autocrine interaction have also been reported (Sierra and Tsao, 2011; Gherardi et al., 2012). All these eventually lead to oncogenic activation of c-Met. A previous study showed that overexpression of c-Met in mice led to eventual spontaneous development of HCC while inactivation of the transgene was found to initiate tumor regression (Wang et al., 2001). Studies have also shown that more c-Met mutations can be found at the site of metastasis, which indicates that such mutations are closely related to tumor progression (Parikh et al., 2014). In patients with gastroesophageal or CRCs, incidence of c-Met mutations is only 1–2% and 2–5% (Cancer Genome Atlas Network, 2012; Dulak et al., 2013; Cancer Genome Atlas Research Network, 2014). Amplification of the c-Met gene also accounts for a small proportion of gastric (3–6%) and CRCs (0.5–2%) (Cancer Genome Atlas Network, 2012; Cancer Genome Atlas Research Network, 2014; Gavine et al., 2015). Functionally, this amplification leads to overexpression of the c-Met protein, which brings about worse prognosis of the disease. Particularly, more c-Met gene amplifications can be found in patients with metastatic liver cancer (Zeng et al., 2008). A large number of solid tumors have been found to abundantly express HGF and c-Met, and this may be due to paracrine and autocrine interaction. *In vivo* studies have provided direct evidence that the effect of autocrine HGF/c-Met signaling is very important for proliferation and growth of lung adenocarcinoma cells (Yi and Tsao, 2000). In addition, expression of HGF can be detected all over the body. A study has shown that HGF is a systemic cytokine that also comes from tumor stroma (paracrine mode) (Sierra and Tsao, 2011). Another possible mechanism of c-Met activation is the crosstalk with other signaling pathways. For instance, while studying HCC, researchers showed that HGF triggered activation of c-Met, which resulted in simultaneous phosphorylation and expression of Caveolin1 (an integral membrane protein involved in signal transduction), while overexpression of Caveolin1 promoted the c-Met signaling pathway (Korhan et al., 2014). Similarly, EGFR stimulation also leads to signal transduction downstream of the c-Met pathway in bladder carcinoma cells that show moderate levels of EGFR and c-Met expression (Yamamoto et al., 2006). Some researchers found that insulin-like growth factor-I (IGF-I)-mediated progression of pancreatic cancer cells depended on c-Met, and the down-regulation of c-Met almost completely inhibited the tumorigenic effect of IGF-I (Yang et al., 2020). Several studies have shown that crosstalk also exists between c-Met and other receptor tyrosine kinase family members such as ERBB2 (also called HER2) and AXL (Khoury et al., 2005; Salian-Mehta et al., 2013). In general, these studies demonstrate that c-Met signaling can be activated in many ways. In non-small cell lung cancer (NSCLC), MET exon 14 (METex14) alterations are considered to be the primary driving mechanism of tumorigenesis. These alterations are closely related to MET overexpression and oncogenesis (Salgia et al., 2020). Previous data have shown that METex14 alterations can be detected in 3–4% of lung adenocarcinoma and 20–30% of pulmonary sarcomatoid carcinomas (Drilon et al., 2017). To date, there are few reports about the METex14 alterations in digestive system cancer. One study examined 230 solid tumor specimens (including 42 gastric and 43 colon cancer specimens) and found METex14 alterations in three gastric samples (7.1%) and four colon cancer samples (9.3%) (Lee et al., 2015). In addition, all the samples of positive METex14 were accompanied with overexpression of c-Met protein. This study preliminarily proved that METex14 alterations might play a driving role in digestive system cancer.

## HGF/C-Met Signaling in Digestive System Cancers

### HGF/c-Met Signaling Inhibitors in Digestive System Cancer

According to different mechanisms and structures, HGF/c-Met axis inhibitors are categorized into anti-HGF and anti-c-Met antibodies as well as small molecule c-Met kinase inhibitors. Based on chemical structures and different docking sites with kinases, there are three types of small-molecule c-Met kinase inhibitors, including selective, non-selective, and special structure c-Met inhibitors (Parikh and Ghate, 2018). So far, most of the HGF/c-Met inhibitors in digestive system cancer have been assessed in preclinical studies (Table 1) and phase I/II/III clinical trials (Table 2).

**TABLE 1:** HGF/c-Met signaling inhibitors in preclinical studies.

Cancer	Agent	Cell lines	Primary results	References
Hepatocellular carcinoma	Cabozantinib	MHCC97H	Cabozantinib inhibited tumor growth by decreasing angiogenesis, inhibiting proliferation, and promoting apoptosis	Xiang et al., 2014
Gastric cancer	Savolitinib	Hs746t	Volitinib displayed a highly selective profile across a gastric cell line panel, potently inhibiting cell growth only in those lines with dysregulated c-Met	Gavine et al., 2015
Pancreatic cancer	Crizotinib	Suit-2	Crizotinib inhibits the peritoneal dissemination of Suit-2 cells	Takiguchi et al., 2017
	Crizotinib + gemcitabine	Orthotopic PDAC-FM-GC mouse models	Crizotinib decreased tumor dimension, prolonged survival, and increased blood and tissue concentrations of gemcitabine	Avan et al., 2013
Melanoma	SU11274	Human malignant melanoma cell lines A375 (ATCC CRL-1619), M14 and M4Beu	SU11274 substantially decreased number of cells in adherent and spheroid cultures, but increased their tumorigenic potential	Kucerova et al., 2016

**TABLE 2:** Summary of HGF/c-Met signaling inhibitors in clinical trials.

Inhibitor name	Targets of inhibitor	Cancer type	Phase	Status	Clinical trial no.
**Anti HGF antibody**					
Rilotumumab (AMG 102)	HGF	Gastric cancer	Phase III	Terminated	NCT02137343
		Gastric cancer	Phase III	Terminated	NCT01697072
		Gastroesophageal adenocarcinoma	Phase II	Unknown	NCT01443065
		Colorectal and gastrointestinal cancer	Phase I/II	Completed	NCT00788957
		Gastric or esophagogastric junction cancer	Phase I/II	Completed	NCT00719550
		Gastric or GEJ cancer	Phase I	Completed	NCT01791374
Ficlatuzumab (AV299)	HGF	Pancreatic cancer	Phase I	Recruiting	NCT03316599
**Anti c-Met antibody**					
Onartuzumab (MetMAb)	c-Met	Gastric cancer	Phase III	Completed	NCT01662869
		Colorectal cancer	Phase II	Completed	NCT01418222
		Gastric cancer	Phase II	Completed	NCT01590719
		Hepatocellular carcinoma	Phase I	Completed	NCT01897038
Emibetuzumab	c-Met	Advanced cancer	Phase I/II	Completed	NCT02082210
Telisotuzumab–Vedotin	c-Met	Advanced solid tumors	Phase I	Recruiting	NCT02099058
**Small molecule c-Met kinase inhibitors**					

***Non-selective c-Met inhibitors (ATP competitive)***					

Crizotinib	c-Met, ALK and ROS1	c-Met positive gastric cancer	Phase II	Completed	NCT02435108
		Solid tumor and colorectal cancer	Phase I	Active, not recruiting	NCT02510001
		Diffuse gastric cancer or breast carcinoma	Phase II	Recruiting	NCT03620643
Cabozantinib	c-Met, VEGFRs, RET, KIT and AXL	Hepatocellular carcinoma	Phase IV	Recruiting	NCT03963206
		Hepatocellular carcinoma	Phase III	Recruiting	NCT03755791
		Colorectal cancer	Phase II	Active, not recruiting	NCT03542877
		Colorectal cancer	Phase I	Completed	NCT02008383
		Pancreatic cancer	Phase I	Completed	NCT01663272
		Pancreatic adenocarcinoma	Phase II	Recruiting	NCT03213626
Foretinib	c-Met, AXL, RON, VEGFR2 and TIE-2	Gastric cancer	Phase II	Completed	NCT00725712
		Hepatocellular carcinoma	Phase I	Completed	NCT00920192
Golvatinib (E7050)	c-Met, VEGFR-2	Hepatocellular carcinoma	Phase I/II	Completed	NCT01271504
		Gastric cancer and solid tumors	Phase I/II	Terminated	NCT01355302
		Gastric cancer and solid tumors	Phase I	Completed	NCT01428141
***Selective c-Met inhibitors (ATP competitive)***					
AMG 337	c-Met	Stomach neoplasms	Phase II	Terminated	NCT02016534
			Phase I/II	Completed	NCT02096666
Volitinib (Savolitinib)	c-Met	MET amplification gastric adenocarcinoma	Phase II	Recruiting	NCT02449551
		MET amplified metastatic or unresectable colorectal cancer	Phase II	Recruiting	NCT03592641
		Gastric adenocarcinoma with c-Met overexpression	Phase II	Recruiting	NCT02447380
		Gastric cancer	Phase I	Completed	NCT02252913
Tepotinib (MSC2156119J)	c-Met	Hepatocellular carcinoma	Phase I/II	Completed	NCT02115373
		Hepatocellular carcinoma	Phase I/II	Active, not recruiting	NCT01988493
Capmatinib	c-Met	Hepatocellular carcinoma	Phase II	Active, not recruiting	NCT01737827
		Hepatocellular carcinoma	Phase I/II	Active, not recruiting	NCT02795429
***Special structure c-Met inhibitors (Non-ATP competitive)***					
Tivantinib (ARQ-197)	c-Met	Inoperable hepatocellular carcinoma	Phase III	Completed	NCT01755767
		Hepatocellular carcinoma	Phase III	Completed	NCT02029157
		Unresectable hepatocellular carcinoma	Phase II	Completed	NCT00988741
		Gastric cancer	Phase II	Completed	NCT01152645
		Pancreatic neoplasms	Phase II	Completed	NCT00558207
		Gastroesophageal cancer	Phase I/II	Completed	NCT01611857
		Hepatocellular carcinoma	Phase I	Completed	NCT00802555
		Advanced hepatocellular carcinoma	Phase I	Completed	NCT01656265

### HGF/c-Met Signaling in HCC

Primary liver cancer is one of the most common malignant tumors in clinics. The condition ranks sixth and second based on incidence and mortality, respectively (Siegel and Miller, 2019). About 75–85% of primary liver cancer cases are HCC (Siegel and Miller, 2019). Sorafenib [a small-molecule multi-kinase inhibitor that targets vascular endothelial growth factor receptors (VEGFRs) rapidly accelerated fibrosarcoma (RAF) and other protein kinases] has been regarded as the standard of treatment for patients with advanced HCC since 2007 (Marisi et al., 2018). However, survival among patients even after treatment with sorafenib is still low, necessitating urgent development of new and effective treatment methods. Studies have demonstrated the role of HGF/c-Met axis in cell survival, cancer proliferation, and metastasis in HCC cases (Bahrami et al., 2017; García-Vilas and Medina, 2018; Ogunwobi et al., 2019) making anti-HGF/c-Met a promising therapeutic target for HCC treatment.

Tivantinib, a staurosporine derivative, is a non-ATP competitive selective small-molecule inhibitor and acts mainly as an anti c-Met inhibitor (Munshi et al., 2010). Previous studies that have targeted the molecule have, in turn, reported encouraging results in several phase I/II clinical trials. For instance, a randomized, controlled, phase II clinical trial evaluated the efficacy of tivantinib in patients with advanced HCC and Child–Pugh A cirrhosis with the results indicating that patients treated with the drug had a longer median disease progression time relative to the placebo (Santoro et al., 2013). Among patients with high c-Met expression, a more significant (2.7 months vs. 1.4 months; *P* = 0.03) curative effect was recorded (Santoro et al., 2013). However, some serious complications recorded in the tivantinib group included anemia and neutropenia, especially when a high dosage was administered. Unfortunately, in a phase III trial of METIV-HCC, tivantinib did not improve overall patient survival compared to the placebo (Rimassa et al., 2018). Nevertheless, there is evidence that a high expression of c-Met in HCC is related to a poor prognosis (Rimassa et al., 2016). Since the METIV-HCC trial selected patients with high c-Met levels, this does not necessarily mean that tivantinib has no role in targeted treatment in HCC patients. Therefore, determination of an accurate dosage coupled with guided patient stratification is key to the use of tivantinib as a target drug for HCC. Some scholars have pointed out that tivantinib cannot inhibit the c-Met tyrosine autophosphorylation, and its biological activity is mainly antimitotic. It kills any kind of cells regardless of the expression of c-Met protein, and the cytotoxicity of tivantinib is related to inhibit microtubule assembly (Basilico et al., 2013). This novel viewpoint may explain the results of phase III clinical trials.

Cabozantinib is a novel small-molecule multi-target tyrosine kinase inhibitor of c-Met, VEGFRs, RET, KIT, and AXL (Yakes et al., 2011). In mice xenotransplantation models, cabozantinib treatment has been shown to inhibit the growth and metastasis of HCC (Xiang et al., 2014). In a phase II placebo-controlled randomized discontinuation study, cabozantinib suppressed tumor growth, disease stability and reduced alpha fetoprotein (AFP) in HCC (Kelley et al., 2017). Recently, a phase III study assessment of the effect of cabozantinib on advanced HCC patients who had been previously treated showed a longer total survival time taken in the cabozantinib group compared to the controls (10.2 vs. 8 months; *p* = 0.005) (Abou-Alfa et al., 2018). The progression-free survival (PFS) period (5.2 vs. 1.9 months; *p* < 0.001) and objective response rate (ORR) (4 vs. < 1%; *p* = 0.009) also improved significantly. The main adverse events were erythrodysesthesia, hypertension, increased aspartate aminotransferase level, fatigue, and diarrhea.

Foretinib is a multi-target inhibitor for c-Met, AXL, RON, VEGFR2, and TIE-2 (Giordano and Columbano, 2014). A phase I/II clinical study evaluating the safety, pharmacodynamics, pharmacokinetics, and activity of foretinib in a late first-line trial reported 30 mg as the maximum dosage for foretinib in phase I. A total of 39 patients received foretinib treatment in phase II (38 patients were evaluable for efficacy), with a median overall survival time (OS) of 15.7 months and a median progression time of 4.2 months reported (Yau et al., 2017). The most common adverse reactions including hypertension (43.6%), loss of appetite (28.2%), ascites (25.6%), and fever (25.6%) were also recorded, although these were not considered serious. Overall, foretinib showed satisfactory antitumor activity in Asia in patients with advanced HCC.

Tepotinib is an effective and highly selective c-Met inhibitor, and its c-Met selectivity exceeds that of most kinase inhibitors with a cell-based 50% inhibitory concentration (IC50) of 1.7 nM (Falchook et al., 2020). In contrast, highly selective c-Met inhibitors have little effect on other targets and are expected to produce less toxicity. Therefore, a sufficient dose can guarantee effective c-Met inhibition. Tepotinib was first approved for the treatment of patients with advanced NSCLC in 2020 in Japan (Markham, 2020). A phase II clinical study on c-Met-positive advanced HCC showed that tepotinib had a longer progression time and PFS time than sorafenib (NCT01988493). The time to progression was 2.9 months in the tepotinib group and 1.4 months in the sorafenib group. The median PFS of the tepotinib group and sorafenib group was 2.8 and 1.4 months, respectively, and the median overall survival was 9.3 and 8.6 months, respectively. Another phase Ib/II study evaluated the efficacy and safety of tepotinib in patients with c-Met-positive HCC who have failed sorafenib treatment, and the PFS rate of 12 weeks was 63.3% (NCT02115373).

Capmatinib (INC280) is a highly selective c-Met inhibitor who has an IC50 of 0.13 nM for c-Met (Bouattour et al., 2018). A phase II study indicated that capmatinib is beneficial to tumor suppression in patients with c-Met-high HCC (Qin et al., 2019). The overall response rate was 30.0% and the disease control rate (DCR) was 50.0% in the c-Met high expression group. It even includes a long-term complete response of more than 600 days and two partial responses. Another phase Ib/II clinical trial is studying the safety and antitumor activity of capmatinib combined with PDR001 [a programmed death 1 (PD-1) inhibitor] in patients with HCC (NCT02795429). The clinical trial is anticipated to be completed at the end of 2020.

Several other similar c-Met inhibitors, such as golvatinib (E7050) and SU11274 have also been reported to have antitumor activity against HCC (Nakagawa et al., 2010; Inagaki et al., 2011). However, studies in relation to their effects in early clinical stages as well as their efficacy and safety are yet to be carried out. In view of the limited therapeutic effect of targeted drugs, a combination of therapies may be a more effective strategy for HCC treatment.

### HGF/c-Met Signaling in Pancreatic Cancer

Pancreatic cancer (PC) is a highly malignant disease with the worst prognosis due to its strong invasion and metastasis ability. The disease’s 5-year survival rate is approximately 9%, the lowest of any cancer (Siegel and Miller, 2019). Pancreatic cancer patients with high c-Met and HGF expression levels tend to show poor prognosis and low survival rates (Yan et al., 2014). Studies have shown that c-Met expression levels in pancreatic tumors are related to tumor grades (Gardian et al., 2012). Similarly, it has been demonstrated that c-Met expression increased in pancreatic ductal adenocarcinoma, suggesting that c-Met might be a molecular marker for predicting prognosis in patients with pancreatic cancer (Zhu et al., 2011).

Crizotinib is an ATP competitive multi-target protein kinase inhibitor that targets c-Met, ALK, and ROS1 (Takiguchi et al., 2017). In an orthotopic mouse model experiment, crizotinib was found to increase the concentration of gemcitabine in blood and tissues, reduce sizes of tumors, inhibit peritoneal dissemination of highly Met-expressing pancreatic cancer, and prolong survival (Avan et al., 2013). Similarly, an *in vitro* experiment showed that crizotinib can be used to treat peritoneal spread of pancreatic cancer based on inhibition of cell proliferation and metastasis mediated by phosphorylation of the c-Met pathway (Takiguchi et al., 2017). However, it is not clear whether the above effects were mediated by the c-Met signaling pathway.

Reports have also indicated that long-term treatments involving cabozantinib induce less resistance and can improve the efficacy of gemcitabine and can overcome gemcitabine resistance in pancreatic cancer (Hage et al., 2013). The effects are achieved by suppressing total c-MET, controlling downstream phosphorylation of c-MET, and decreasing expression of transcription factor SRY-related HMG-box 2 (SOX2) (Hage et al., 2013). An experiment conducted to determine maximum tolerable concentrations of cabozantinib and gemcitabine in 10 advanced pancreatic cancer patients showed that more than 25% of the patients in all concentration groups had dose-limiting toxicity (≥grade 3 ALT or AST elevations or ≥grade 3 thrombocytopenia) (Zhen et al., 2016). Despite the small sample size used in the study, a certain level of significance for future research of cabozantinib can be still be derived from the findings.

### HGF/c-Met Signaling in Gastric Cancer

Gastric cancer is the fifth most common form of cancer worldwide and the third leading cause of cancer-related deaths (Padmanabhan et al., 2017). Gastric cancer patients who have a high expression of c-Met show poor prognosis compared to those with c-Met negative tumors (Fuse et al., 2016). Numerous preclinical and clinical studies have generated data on monoclonal antibodies against HGF/c-Met axis, which provide possible targets for development of gastric cancer treatments.

Rilotumumab (AMG102), a monoclonal antibody of the human IgG2 targeting HGF, can block binding of HGF to c-Met. A randomized phase II clinical trial showed that this antibody in combination with epirubicin, cisplatin, and capecitabine (ECX) could prolong PFS in advanced gastric cancer patients relative to the control group, especially in patients with high c-Met expression (Iveson et al., 2014). Based on these results, a further study on the effects of rilotumumab combined with ECX in treatment of gastric cancer was conducted using two phase III trials (RILOMET-1 and 2) (Doi et al., 2015; Catenacci et al., 2017). Unfortunately, both trials were terminated because RILOMET-1 results showed an increase in the number of deaths due to complications in patients treated with rilotumumab compared to the placebo (Catenacci et al., 2017).

Onartuzumab is a recombinant humanized anti-c-Met monoclonal antibody, which is produced in *Escherichia coli* (Merchant et al., 2013). Evaluation of the safety and efficacy of onartuzumab combined with mFOLFOX6 in patients with HER2-negative gastric cancer showed that adding onartuzumab to mFOLFOX6 failed to improve efficacy of c-Met immunohistochemically positive population or even the whole population (Shah et al., 2016). Similarly, a phase III clinical trial revealed no significant improvements in clinical benefits in first-line chemotherapy for HER2-negative and c-Met-positive advanced gastroesophageal adenocarcinoma (GEC). Another small molecule, AMG 337, which is a selective inhibitor targeting c-Met has also been reported (Hughes et al., 2016). A phase II clinical study assessing 45 patients with gastric/gastroesophageal junction/esophageal tumor and AMG 337 monotherapy patients diagnosed with c-Met-amplified tumors showed an ORR of 18%, indicating that AMG 337 has certain antitumor activities (Van Cutsem et al., 2019).

Emibetuzumab (LY2875358) is a humanized immunoglobulin G4 monoclonal anti-Met antibody. The drug inhibits the activation of HGF/c-Met pathway by preventing HGF from binding to its receptor, c-Met, and by degrading c-Met (Liu et al., 2014). A phase II study evaluated the safety and efficacy of emibetuzumab in patients with advanced gastric cancer. The 8-week PFS rate is 47%, and the DCR is 40%. Although the sample size is small, monotherapy of emibetuzumab was well tolerated and showed certain anti-tumor activity (Sakai et al., 2017). A phase Ib/II study evaluated the efficacy of emibetuzumab combined with ramucirumab (a monoclonal anti-VEGFR-2 antibody) in 97 patients with solid tumors (including 16 gastric or gastroesophageal junction adenocarcinoma, 45 HCC). The results showed that the combination therapy had more significant antitumor activity.

Telisotuzumab (ABT-700) is a novel anti-c-Met antibody that binds c-Met with high affinity and inhibits c-Met signaling. Unlike most other c-Met antibodies, it destroys the productive dimerization and activation induced by HGF or c-Met on the cell surface independent of ligand (Wang et al., 2016). A phase I study evaluated the safety and efficacy of telisotuzumab in patients with advanced solid tumors. However, significant clinical antitumor activity was only observed in patients with MET-amplified gastroesophageal cancer (Strickler et al., 2020). Considering that primary MET genomic amplification a low-frequency event in most tumors, the researchers developed an antibody–drug conjugate, telisotuzumab–vedotin, also known as ABBV-399, which is composed of telisotuzumab and cytotoxic monomethyl auristatin E (MMAE) via a valine–citrulline linker (Wang et al., 2017). Telisotuzumab–vedotin does not require the tumors to be addicted to the oncogene. It delivers cytotoxin MMAE directly to c-Met-positive tumor cells and has demonstrated antitumor activity in tumors without increased copy number of the MET gene (Strickler et al., 2018). A phase I clinical trial is studying the safety and preliminary efficacy of telisotuzumab–vedotin in patients with advanced solid tumors (NCT02099058). The clinical trial is anticipated to be completed at the beginning of 2022.

Savolitinib is a novel and highly selective c-Met inhibitor, which has shown encouraging results in the phase III randomized clinical trial of papillary renal cell carcinoma (PRCC) (Choueiri et al., 2020). In a preclinical study, savolitinib showed tolerable side effects and significant antitumor effect in c-Met-amplified mice (Gavine et al., 2015). Recently, a multiple-arm clinical trial evaluated the efficacy of targeted therapy in 772 patients with metastatic gastric cancer (Lee et al., 2019). The results showed that there was the highest response rate in the fourth arm (c-Met amplification–savolitinib monotherapy). One patient developed malignant ascites after failure of capecitabine/oxaliplatin treatment. Following savolitinib treatment, the tumor was significantly reduced and the patient achieved curative resection.

Tivantinib, foretinib, and other c-Met tyrosine kinase inhibitors (TKIs), have previously resulted in no clear antitumor activity in gastric cancer patients (Shah et al., 2013; Kang et al., 2014). Although results from several recent clinical trials do not show promise as anti-c-Met drugs, a combined therapeutic strategy for multiple downstream signal transduction could have clinical potential, especially in c-Met-positive patients.

### The Role of HGF/c-Met Signaling in Colorectal Cancer

Colorectal cancer is the third most common cancer in the world, although a decline in CRC mortality has been observed in the recent past (Siegel and Miller, 2019). Various studies have reported that overexpression or amplification of c-Met in early (I and II) and late CRC (III and IV) is closely related to its invasion and distant metastasis (Zeng et al., 2008; Garouniatis et al., 2013; Lee et al., 2018). For this reason, studies have hypothesized that inhibition of c-Met activation may decrease tumor activity in CRC.

Ficlatuzumab (AV-299) is a humanized, high-affinity monoclonal antibody that can block HGF/c-Met binding and mediate downstream phosphorylation of the signaling pathway (Mok et al., 2016). A study assessing tolerability and safety of ficlatuzumab in advanced CRC patients demonstrated that the antibody has the ability to regulate HGF/c-Met pathway and downstream signaling (Tabernero et al., 2014).

The therapeutic effect of rilotumumab in CRC has also been evaluated in a randomized phase Ib/II research where panitumumab (a monoclonal antibody targeting EGFR) was combined with rilotumumab or placebo in patients with kirsten rat sarcoma (KRAS) wild-type metastatic CRC who met the prespecified criterion for improvement in ORR (Van Cutsem et al., 2014). Although the combined inhibition of HGF/c-Met and EGFR showed some encouraging results, no further research findings have been reported with regard to rilotumumab and panitumumab development in CRC.

Another new compound, SU11274, that targets the c-Met ATP-binding site has also been reported and implicated in blocking of HGF-dependent c-Met activation (Gao W. et al., 2013). *In vivo* experiments showed that daily administration of SU11274 in mice resulted in inhibition of tumor growth in xenografts. It was also found that SU11274 could significantly inhibit survival and growth of colon cancer cells (Gao S. H. et al., 2013). However, a study of melanoma *in vitro* showed that the off-target action of SU11274 resulted in the increase of tumorigenic potential (Kucerova et al., 2016). Whether SU11274 can be used as a candidate drug to inhibit c-Met necessitates further clinical trials.

Analysis of clinical effects of tivantinib, following its gradual introduction into CRC, reveals negative results. For instance, a randomized controlled phase I/II clinical trial of tivantinib combined with irinotecan and cetuximab showed no significant improvement of PFS or OS in metastatic CRC patients with wild-type KRAS (Eng et al., 2016). Similarly, a new multicenter phase II study suggested that a combination of tivantinib and cetuximab did not achieve the expected efficacy in CRC patients who had high c-Met expression and acquired resistance to anti-EGFRs (Rimassa et al., 2019). However, a study indicated that inhibition of the HGF/c-MET pathway can improve the sensitivity of CRC to EGFR inhibitors, indicating that combination therapy could still be a future research direction (Liska et al., 2011).

Currently, relatively few reports about HGF inhibitors in CRC exist. In general, though the research on anti-HGF/c-Met axis for CRC is still in its infancy, they have great potential in the future treatment.

### HGF/c-Met Signaling in Non-digestive System Cancer

In addition to digestive system cancers, the study of c-Met inhibitors is also very active for other cancer. A large number of clinical trials have been carried out on melanoma, breast cancer, PRCC, NSCLC, and medullary thyroid cancer (MTC), providing a lot of reliable experience for the treatment of digestive system cancers (Table 3).

**TABLE 3:** The clinical research of c-Met inhibitors in non-digestive system cancers.

Cancer	Agents	Phase	Primary results	References
Melanoma	Cabozantinib	II	Cabozantinib vs. temozolomide or dacarbazine: PFS: 60 vs. 59 days (*P* = 0.964; HR = 0.99). OS: 6.4 vs. 7.3 months (*P* = 0.580; HR = 1.21). Cabozantinib demonstrated no improvement in PFS but an increase in toxicity.	Luke et al., 2020
Breast cancer with bone metastases	Cabozantinib	II	Bone scans improved in 38% of patients and remained stable in an additional 12% for a minimum duration of 12 weeks. PFS was 4.3 months and OS was 19.6 months.	Xu et al., 2020
Papillary renal cell carcinoma	Savolitinib vs. sunitinib	III	PFS: 7.0 months (95% CI, 2.8–not calculated) for savolitinib and 5.6 months (95% CI, 4.1–6.9) for sunitinib. Savolitinib demonstrated encouraging efficacy with fewer grade 3 or higher adverse events.	Choueiri et al., 2020
Non-Small Cell Lung Cancer	Tepotinib	II	The response rate of liquid-biopsy group (*n* = 66) and tissue-biopsy group (*n* = 60) were 48% and 50%, respectively. Median duration of response was 11.1 months.	Paik et al., 2020
	Tivantinib + erlotinib	III	Erlotinib + tivantinib vs. erlotinib + placebo; PFS: 13.0 vs. 7.5 months; OS: 25.5 vs. 20.3 months. Erlotinib + tivantinib was tolerable and showed improved efficacy over erlotinib monotherapy.	Scagliotti et al., 2018
Medullary thyroid cancer	Cabozantinib	III	Cabozantinib vs. placebo: PFS: 11.2 vs. 4.0 months; ORR: 28 vs. 0%.	Elisei et al., 2013

A phase II clinical trial evaluated the efficacy of cabozantinib compared with traditional chemotherapy in the treatment of uveal melanoma (Luke et al., 2020). The result showed that cabozantinib did not demonstrate improvement in PFS (cabozantinib vs. temozolomide or dacarbazine: 60 vs. 59 days). In another phase II study, cabozantinib showed bone-centric activity in patients of breast cancer with bone metastases (Xu et al., 2020). Savolitinib has shown encouraging results in the phase III randomized clinical trial of PRCC (Choueiri et al., 2020). In this clinical trial, 60 patients were randomly divided into group savolitinib (*n* = 33) and group sunitinib (*n* = 27). The primary end point was PFS; group savolitinib and group sunitinib were 7.0 and 5.8 months, respectively. Tivantinib and tepotinib have demonstrated significant antitumor activity in NSCLC. In a phase III study of tivantinib in patients with NSCLC, tivantinib combined with erlotinib led to a significant prolongation of PFS (13.0 vs. 7.5 months) and OS (25.5 vs. 20.3 months) compared to erlotinib monotherapy (Scagliotti et al., 2018). A phase II study evaluated the efficacy of tepotinib in patients with advanced NSCLC with METex14 alterations (Paik et al., 2020). The response rate of the liquid-biopsy group (*n* = 66) and the tissue-biopsy group (*n* = 60) were 48% and 50%, respectively. Median duration of response was 11.1 months. In a phase III clinical trial, 330 patients with progressive metastatic MTC were randomly assigned to the cabozantinib group (*n* = 220) or the placebo group (*n* = 110) (Elisei et al., 2013). Median duration of PFS was 11.2 and 4.0 months in cabozantinib and placebo, respectively. ORR was 28% in the cabozantinib group and 0% in the placebo group. The therapeutic effect of cabozantinib on MTC was statistically significant.

## Prognostic Effect of HGF/C-Met Signaling Pathway in Digestive System Cancer

Previous clinical trials have indicated that that high expression of c-Met gene is involved in poor prognosis and high risk of many types of cancer. Consequently, scientists have extensively studied the relationship between overexpression of the c-Met gene and prognosis of a single tumor, mainly focusing on CRC, gastric cancer, breast cancer, and lung cancer (Bradley et al., 2018). A meta-analysis of 11 retrospective studies (including 1895 patients with CRC) suggested that OS (HR 1.33, 95% CI 1.06–1.59) and PFS duration (HR 1.47, 95% CI 1.03–1.91) in patients with positive c-Met was low (Liu et al., 2015). Similarly, another meta-analysis of 14 retrospective studies (including 2258 patients with gastric cancer) showed that high amplification and expression of the c-Met gene in gastric cancer is significantly related to poor prognosis (Peng et al., 2014). Furthermore, a meta-analysis of 1408 postoperative patients with liver cancer revealed a significant reduction in the relapse-free survival (HR 1.26; 95% CI, 1.02–1.56) and total survival (HR 1.16; 95% CI, 1.03–1.31) rates of patients overexpressing c-Met (Kim et al., 2017a). Similar studies have been carried out among patients diagnosed with pancreatic, biliary tract, esophageal, as well as other types of cancer (Kim et al., 2017b; Ren et al., 2017; Zhang Z. et al., 2018). All these results show that overexpression of the c-Met gene is significantly correlated with and can lead to the poor prognosis of these tumors.

## Limitations of the Efficacy of C-Met Inhibitors

A large number of clinical trials have proved that the safety of HGF or c-Met inhibitors is reliable; however, there is no significant clinical benefit for patients. A variety of potential factors contribute to the regrettable result. The pathway of c-Met signal transduction is extremely complex, and there is extensive crosstalk between c-Met and other carcinogenic pathways, which may lead to the treatment of targeting HGF, or c-Met cannot fully block the downstream signal transduction. In addition, the inhibition of c-Met promotes the stability of PDL1 and makes tumor cells escape from immune surveillance. A study found that the expression of PDL1 is up-regulated in the HCC cells of mice treated with c-Met inhibitors, which induces the functional inactivation of T-cells and enables HCC cells to escape from the killing of T-cells (Li et al., 2019). Similar results were observed in an *in vitro* experiment of NSCLC (Sun et al., 2020). Therefore, the monotherapy of targeting c-Met may not bring significant clinical benefits to patients. Adopting novel combination strategy is the key of the research.

## Combination Therapy

A study has shown that inhibiting the c-Met signaling pathway compensatively activates the EGFR pathway (Steinway et al., 2015). In addition, MET amplification is one of the mechanisms of drug resistance of EGFR TKI (Kumar et al., 2020). Therefore, combination therapy strategy targeting the c-Met and EGFR pathway may bring more significant anti-tumor effect. In particular, several studies have shown significant benefits of the combination therapy in NSCLC, melanoma, breast cancer, CRC, and HCC (Steinway et al., 2015; Zielonka et al., 2015; Dratkiewicz et al., 2019; Rimassa et al., 2019; Chu et al., 2020). An *in vitro* study showed that simultaneous inhibition of c-Met and EGFR pathways inhibits HCC tumor growth (Steinway et al., 2015). Similarly, the combined elimination of c-Met and EGFR significantly inhibited the proliferation of hepatocytes (Bhushan et al., 2019). A phase Ib study of patients with MET-positive CRC indicated that the treatment strategy of capmatinib in combination with cetuximab (an anti-EGFR monoclonal antibody) was tolerable and showed preliminary antitumor effects (Delord et al., 2020). Overall, the combined inhibition of c-Met and EGFR is a promising treatment strategy, warranting further exploration.

A study has indicated that the anti-tumor effect of the single drug of tivantinib or anti-PD1 is not as good as the combination of tivantinib and anti-PD1, and the combined treatment is more effective in inhibiting the growth of liver tumor (Li et al., 2019). Similarly, another study showed that anti-PD1 treatment can inhibit the expression of PDL1 induced by c-Met inhibitors, and the combination of c-Met inhibitor and anti-PD1 can better control the tumor progression (Glodde et al., 2017). A clinical case report evaluated a patient with advanced HCC. The patient was given 60 mg cabozantinib daily. One month later, nivolumab (100 mg per 21 days), a PD1 inhibitor, was added to the patient’s treatment regimen, and the patient showed a good tolerance response. Surprisingly, after the treatment of cabozantinib and nivolumab, the patient achieved a PFS of more than 25 months (Yang et al., 2020). A large number of clinical trials have been carried out to evaluate the combination regimens of c-Met inhibitors, anti-PD1 agents, and other drugs in the treatment of malignant tumors. For example, the combination of cabozantinib plus nivolumab is now being evaluated in a phase I study of patients with advanced HCC, and the primary endpoints are feasibility and efficacy (NCT03299946). Similarly, a clinical study is evaluating cabozantinib in combination with durvalumab (a PD1 inhibitor) in patients with advanced gastroesophageal cancer and other gastrointestinal malignancies (NCT03539822). Most noteworthy, the combination of cabozantinib and atezolizumab (a PD-L1 inhibitor) is now being evaluated in a phase III study of patients with advanced HCC (NCT03755791). The primary objectives of this study are OS and PFS. Experimental arm receives cabozantinib 40 mg once a day, plus atezolizumab 1200 mg i.v. every 3 weeks. Control arm will receive sorafenib 400 mg twice a day. The clinical study is anticipated to be completed at the end of 2021. In addition, the efficacy and safety of cabozantinib + nivolumab for patients with advanced HCC were first presented at 2020 ASCO Gastrointestinal Cancer Symposium. A total of 71 patients were included in the study. Thirty-five patients were treated with cabozantinib + nivolumab (doublet therapy) and 36 patients were treated with cabozantinib + nivolumab + ipilimumab (an antibody inhibiting CLTA-4) (triple therapy). ORR was 17% and 26% in the doublet therapy and triple therapy groups, respectively. DCR of the two groups was 81% and 83% and the median PFS was 5.5 months and 6.8 months, respectively. The clinically meaningful responses from these cabozantinib combinations are encouraging. Overall, these clinical studies likely portend the future of gastrointestinal tumors treatment.

## Conclusion

An imbalance in HGF/c-Met signal transduction is a driving factor for cancers of the digestive system, and this promotes tumor growth, as well as its invasiveness and dissemination. Numerous studies have shown the wide application of c-Met in digestive system cancers with related clinical and preclinical evidences supporting the anti-HGF/c-Met signaling pathway as a reliable treatment for HCC, gastric, pancreatic, and CRC among others. In particular, c-Met-positive patients showed encouraging results in the phase II study, suggesting that finding the most appropriate subgroup of patients may make the most of HGF inhibitors. A crosstalk between c-Met and other signaling channels holds the key to future therapies. Particularly, single targets for inhibition of HGF/c-Met may not be the most ideal method for developing treatment against this condition. It will also be important for future research works to target other key signaling pathways during development of therapies.

In general, the HGF/c-Met axis is an excellent therapeutic target, but it needs a more in-depth and guided research approach. The key challenges for future research include the selection of patients who may benefit from the treatment, development of optimal therapeutic combinations, and identification of highly sensitive biomarkers for monitoring disease prognosis.

## Author Contributions

ZS, HP, ST, and JZ drafted the manuscript. AS and SY reviewed and modified the manuscript. All authors contributed to the article and approved the submitted version.

## Conflict of Interest

The authors declare that the research was conducted in the absence of any commercial or financial relationships that could be construed as a potential conflict of interest.
